# Variance of *K*_s_ distribution corrects the bias in the divergence caused by the ancestral population size

**DOI:** 10.3389/fgene.2025.1725551

**Published:** 2025-12-17

**Authors:** Mi-Jia Li, Xiao-Xue Li, Lin-Lin Xu, Bo-Wen Zhang

**Affiliations:** 1 Ministry of Education Key Laboratory for Biodiversity Science and Ecological Engineering, College of Life Sciences, Beijing Normal University, Beijing, China; 2 Department of Microbiome Dynamics, Leibniz Institute for Natural Product Research and Infection Biology (Leibniz-HKI), Jena, Germany

**Keywords:** K_s_ distribution, effective population size, species divergence, coalescent model, gene divergence, orthologous gene

## Abstract

$K_{s}$ distribution, the distribution of the synonymous substitutions, has been widely used to estimate the species divergence using orthologous genes. However, conventional approaches often ignore the underlying bias that species divergence is delayed to average gene divergence by 2*N*_e_ generations, where *N*_e_ represents the ancestral effective population size, due to the lack of scalable methods for *N*_e_ inference. Here, we demonstrate through simulations that *K*_s_ distribution variance correlates with *N*_e_, enabling direct estimation of ancestral population parameters from standard *K*_s_ data. Leveraging this relationship, we present *Tspecies*, a framework that corrects divergence time estimates using only substitution rates and *K*_s_ distributions, without requiring additional genomic data. Our practical application of *Tspecies* in *Liriodendron* has inferred a divergence time between North American and East Asian lineages (1.44 Ma) that align with early Pleistocene glaciation, and a large ancestral *N*_e_ (∼5.29 × 10^4^) consistent with fossil evidence. Our finding reveals the correlation between the variance of *K*_s_ distribution and *N*_e_, and develops a computational framework to resolve the bias in *K*_s_ based dating by incorporating a readily estimated *N*_e_.

## Introduction

*K*_s_ distribution, the distribution of the synonymous substitutions of orthologs, has been widely employed to estimate the species divergence times in comparative genomics. When studying the characteristics of some specific genes in *Gossypium hirsutum* (Hao et al., 2020), *Raphanus sativus* (Hu et al., 2018), *Brassica napus* (Zhu et al., 2020), researchers have successfully employed the *K*_s_ distributions to depict the divergence times of the objects from their close relatives. The *K*_s_ value reflects the sum of independent evolutionary distances accumulated in two species following their divergence. Building on this, under the assumption of constant mutation accumulation rates in both species, the divergence time *T* can be calculated by dividing the *K*_s_/2 value (representing the distance from either species to their common ancestor) by the substitution rate (*μ*)*.* This *K*_s_ distribution-based method for estimating species divergence times has been widely adopted in comparative genomic studies and can be easily obtained with the commonly used genomic analysis toolkits such as OrthoFinder (Emms and Kelly, 2019) and KaKs_Calculator (Zhang, 2022).

**FIGURE 1:**
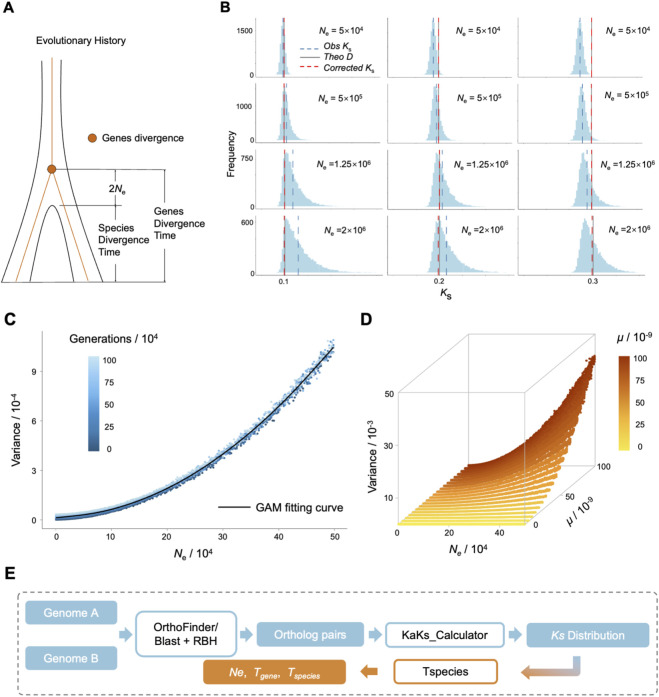
The optimization for the distribution of the synonymous substitutions (*K*_s_) **(A)** Schematic diagram of species divergence time and the average divergence time of genes. **(B)** Observed, theoretical, and corrected *K*_s_ in the *K*_s_ distribution of different simulated scenarios. The *Obs K*_s_ refers to the *K*_s_ directly calculated by the sequential differences; The *Theo D* refers to the theoretical species distance, which equals to twice *μ* multiplied by the simulated species divergence time; The *Corrected K*_s_ refers to the estimated species divergent distance corrected with 2*N*_e_ and multiple substitution. **(C)** The Generalized Additive Model (GAM) describing the relationship between *N*_e_ and variance of *K*_s_ distribution. **(D)** 3D Plot of Variance of *K*_s_ distribution vs. *N*_e_ and *μ*. **(E)** Overview of the workflow utilized by *Tspecies*. Orthologous pairs were inferred through OrthoFinder or blast with reciprocal best hit (blast + RBH) before calculating *Ks* distribution.

However, under the coalescent theory framework, when post-divergence gene flow is not considered, the genetic divergence time between orthologs always predates the species divergence time (Sigwart, 2009). Therefore, accurate estimation of species divergence time requires calculating the difference *dT* between these two temporal scales. And for *dT*, according to the coalescent theory, the species divergence time differs from the average gene divergence time by 2*N*_e_ (Figure 1A), where *N*_e_ represents the ancestral effective population size (Wakeley and Sargsyan, 2009). Current methods for inferring *N*_e_ primarily rely on population genomic data to reconstruct historical demographic dynamics (Charlesworth, 2009). However, due to limitations in sampling or sequencing, most studies lack sufficient population-level genomic data to estimate *N*_e_ (Wang et al., 2016). To address these constraints, alternative approaches that can estimate both species divergence and *N*_e_ using single or few genomes have thus been developed, such as the Bayesian Phylogenetics & Phylogeography (BPP) program (Yang, 2015) and the F1-hybrid Pairwise Sequentially Markovian Coalescent (hPSMC) model (Cahill et al., 2016). While BPP has been widely applied and provides powerful inference under the multispecies coalescent framework, its performance can be limited in practice. For instance, it often requires high-quality genomic data (e.g., haplotype-resolved genomes) and substantial computational resources. Complex evolutionary scenarios such as polyploidy (Yan et al., 2022) or ghost introgression (Pang and Zhang, 2024) can further complicate parameter estimation.

In practice, comparative genomics studies often used this *K*_s_ distribution-based method, instead of utilizing the coalescent theory framework, for estimating species divergence times in comparison with whole genome duplication or other evolution events. Therefore, this *N*_e_-dependent bias is often overlooked. Directly scaling *N*_e_ through *K*_*s*_ distributions could thereby offer a more feasible solution in such cases. Of note, the practical application of our tool on the genomes from genus *Liriodendron* indicated a divergence time of ∼1.44 million years ago (Ma) between the North American and East Asian lineages, which gives a more plausible scenario of allopatric speciation driven by glacial divergence.

## Methods

### Simulating the divergence

To model the divergence of the two closely related species, we employed the *ms* (Hudson, 2002) and the *seq-gen* (Rambaut and Grass, 1997). Given that the samples were generated under neutral model, the rate of nucleotide substitutions rate between sequences was equivalent to the rate of synonymous substitutions. In each simulation, 10,000 pairs of homologous sequences of 1,000 bp in length were generated, and the mean and variance of the *K*_s_ distribution were calculated using R.3.6.0. To account for the range of divergence scenarios, the substitution rate (*μ*) was grouped *μ* into 23 discrete categories spanning 1 × 10^−10^ to 1 × 10^−7^ per site per generation, a range that encompasses empirically reported neutral mutation rates across eukaryotes (Wang and Obbard, 2023). The theoretical divergence time (*T*) was set to 10^4^ to 10^6^ generations, and *N*_e_ was set to 500 to 500,000. These values are covered by *θ* and *t* in *ms*. In this case, *θ* is defined as 4*N*_e_ *μ*, and *t* is the theoretical divergence time divided by four times the effective population size.

### Modeling the *N*_*e*_ effects

We first derived the theoretical relationship between *N*_e_ and the variance of synonymous divergence under the standard coalescent model, yielding the analytical estimator:

$$N_{e}=\sqrt{\frac{var\left(Ks\right)}{16\times \mu^{2}}}$$

Where var is the variance of the *K*_*s*_ distribution, and the *μ* is substitution rate in the unit of per site per generation. This approximation is accurate only when *N*_e_ is sufficiently large, because its validity requires the coalescent variance to dominate the Poisson variance of mutation counts. Based on the empirical evaluation from simulation mentioned above, this condition holds reliably when *N*_e_ > 450,000. In practice, *Tspecies* switches to the analytical coalescent estimate when the predicted value exceeds this threshold.

For smaller effective population sizes, the relationship between *N*_e_ and the variance of *K*_s_ is strongly nonlinear and lacks a convenient analytical approximation. In this regime, we approximate the *N*_e_ -Ks variance relationship using generalized additive models (GAMs). For each mutation rate *μ*, we generated training data with *ms* and *seq-gen*, recording the true *N*_e_ used in the simulations and the resulting variance of *K*_*s*_ across 10,000 simulated loci. We then fitted a GAM of the form *N*_e_ ∼ s (Var(*K*_*s*_)), using gam () function in *mgcv* (Wood, 2017) with default smoothing settings. Mutation rate *μ* was treated as a categorical factor, and *μ*-specific models (and their prediction grids) are included in the *Tspecies* package. During the inference, *Tspecies* uses the GAM-based predictions whenever the estimated *N*_e_ is below 450,000, interpolating Ne from the empirical Var(*K*_*s*_) via the pre-fitted model for the corresponding *μ.*

### Robustness on *N*_e_-*K*_s_ variance relationship

To evaluate the impact of sequential length on the *N*_e_-*K*_s_ variance relationship, we compared model predictions across *L* = 500 bp, 1,000 bp, 1,500 bp and 2,000 bp while holding other parameters constant (*μ* = 10^–8^ per site per generation). For each *L*, we generated orthologous sequence pairs using *ms* and calculated the normalized variance of *K*_s​_ distributions (Supplementary Figure S1).

In order to ascertain whether the predicted outcomes of the *Tspecies* undergo substantial alteration when *N*_e_ fluctuates, a simulation was conducted in *ms*: subsequent to species divergence, the number of one of the subpopulations diminished precipitously to one-half, one-fifth, and one-tenth of the ancestral population, respectively (Supplementary Figure S2). *Tspecies* was utilized to calculate the species divergence times, and the predicted outcomes were compared with the set theoretical times in simulation. Then, the relative accuracy (*RA*) was calculated by the following formula:

$$RA=\frac{\left.\left|T-\right.T_{true}\right|}{T_{true}}$$

where T is species divergence time calculated by *Tspeices*, $T_{true}$ is theoretical species divergence time. The relative accuracy under different *μ* are shown in Supplementary Figure S3.

### Scale generation times to real times

In *Tspecies*, the mutation rate *μ* is specified per site per generation. When the input mutation rate is provided per site per year, it is internally converted to a per-generation rate by multiplying by the generation time *g* (in the unit of years per generation). Similarly, *Tspecies* outputs the divergence time in units of generations; when a generation time *g* is supplied, these values are converted to years by multiplying the estimated number of generations by *g*.

### Application in *liriodendron*

To test the model, we applied *Tspecies* to the genomes of genus *Liriodendron* with two distinct species from East Asian (*L. chinense*) and eastern North American (*L. tulipifera*). Genomes were downloaded from the database PRJNA418360 of NCBI. The *K*_s_ distribution of reciprocal best-hit gene pairs across the genomes was calculated using the *K*_s_ analysis pipeline implemented in the wgd package (Zwaenepoel and Van de Peer, 2019). The obtained divergent distance is scaled with the reported synonymous substitution rate of 3.02 × 10^−9^ per site per year (Cui et al., 2006). The divergence time directly calculated by mean of the *K*_s_ distribution was compared to the time estimated under *Tspecies*. The δ^18^O data from the Lisiecki and Raymo (2005) are converted to direct temperature estimates using the equations of Hansen (Hansen et al., 2013).

### Coalescent-based bayesian analyses

The bpp v4.1.4 (Yang, 2015) was used to calculate the divergence time of East Asian (*L. chinense*) and eastern North American (*L. tulipifera*) under coalescent-based Bayesian analyses. We used A00 model with fixed species branching order to estimate the parameters. The alignments of 1,000 loci with 500 bp length were used, with MCMC chain length of 1,000,000 and the first 100,000 discarded as burn-in. Tau (for divergence time) and Theta (for effective population sizes) parameters were estimated with substitution rate of 3.02 × 10^−9^ per site per year and generation time of 30 years. The ESS for each parameter was confirmed to be larger than 200 in MCMC trace files to guarantee convergence.

## Results

The core concept underlying this optimization is rooted in the coalescent theory. Because the *K*_s_ distribution reflects divergence among orthologous loci from two sister species, these loci coalesce exclusively within the ancestral population. Consequently, the shape of the *K*_*s*_ distribution should conform to the coalescent model of the ancestral lineage, with *N*_e_ representing the ancestral population size. Under this framework, the expected coalescent time for two alleles from a random locus is 2*N*_e_ generations earlier than the species divergence (Figure 1A). Simulation analyses confirmed this theoretical expectation, revealing a critical deviation between *N*_e_ and *K*_s_-based divergence estimates. As illustrated in Figure 1B, direct estimation of species divergence using mean *K*_s_ values exhibited systematic biases proportional to *N*_e_. Moreover, incorporating *N*_e_-correction together with multi-locus substitution models (Nielsen and Slatkin, 2013) substantially reduced these deviations, bringing the corrected estimates into close agreement with the true divergence times.

Under the coalescent model of a constant population with large *N*_e_, the standard deviation for the coalescent time for two alleles is approximately 2*N*_e_. Although explicit species divergence scenarios introduce additional complexity, we hypothesized that the variance of *K*_s_ distributions could serve as a quantitative proxy for *N*_e_. To test this hypothesis, we simulated the species divergence under varying *N*_e_ and examined the resulting relationship between *N*_e_ and *K*_s_ variance. The simulation results (Figure 1C) revealed two salient patterns: (1) a strong positive correlation between *N*_e_ and *K*_s_ variance persisted across all divergence times *T*; (2) at large *N*_e_ (when *N*_e_ > 450,000), the relationship closely followed the analytical expectation, whereas at smaller *N*_e_ all trajectories converged toward a single smooth curve.

To capture the observed *N*_*e*_-*K*_*s*_ variance relationship, we fitted a generalized additive model (GAM). The GAM revealed a strongly nonlinear yet highly predictive relationship between *N*_e_ and *K*_s_ variance (*R* ^2^ = 0.997, Figure 1C). The smooth term for *N*_e_ showed significant complexity (effective degrees of freedom [edf] = 7.11). Model diagnostics confirmed robustness: the intercept (3.68, standard error = 0.0045) remained stable across divergence times (*t* = 815.8), while the nearly identical generalized cross-validation (GCV) score (0.0289) and scale estimate (0.0287) ruled out overfitting. Importantly, this framework retains strong predictive power for estimating *N*_e_ from empirical *K*_s_ distributions, provided that orthologous loci are sufficiently sampled across the genome.

While our model provides a computationally tractable framework to correcting divergence time estimates, it relies on simplifying assumptions, including identical sequence lengths and constant *N*_e_ across speciation events. To evaluate its robustness, we conducted sensitivity analyses under varying substitution rate (*μ*), sequential lengths (*L*) and post-divergence *N*_e_ dynamics. Simulations demonstrate that *μ* significantly modulates the *N*_e_-*K*_s_ variance pattern (Figure 1D). Higher *μ* values intensified the positive correlation between *N*_e_ and *K*_s_ variance, underscoring the necessity of incorporating *μ* as a critical input parameter in our framework. The relative difference in predicted variance in different *L* (Supplementary Figure S1) suggested potential sequence length effects at shorter *L* (e.g., *L* = 500 bp), whereas longer sequences (*L* ≥ 1, 000 bp) produced stable estimates (mean deviation = 12.2%). Additionally, simulations considering post-divergence *N*_e_ dynamics (Supplementary Figure S2) suggested only a minor bias (∼5.6%) from true divergence times, with higher *μ* improving accuracy (Supplementary Figure S3). Together, these results indicate that our proposed framework (Figure 1E) remains robust across a wide range of demographic and mutational scenarios.

**FIGURE 2:**
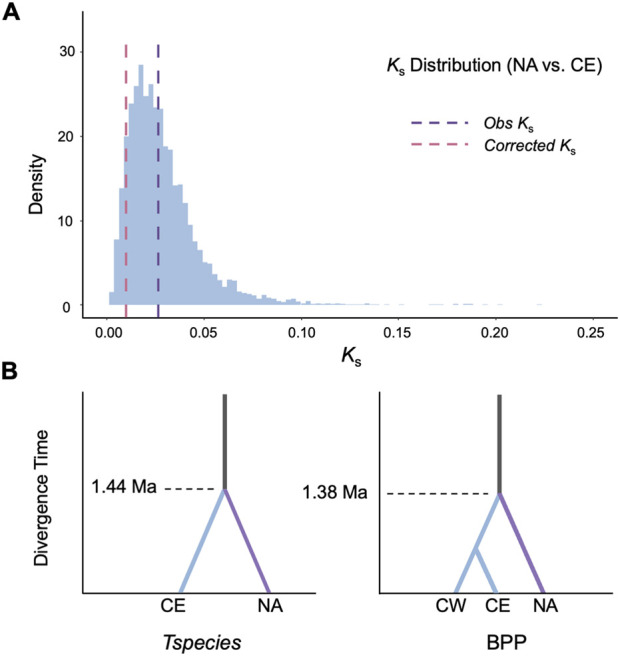
Practical application of *Tspecies* in *Liriodendron*. **(A)** *K*_s_ distribution *(Top)* for orthologs between *L. tulipifera* from North America (NA) and *L. chinense* from eastern China (CE). **(B)** The species divergence time of *L. chinense* and *L. tulipifera* estimated by *Tspecies* and BPP. NA, *L. tulipifera* from North America; CE, *L. chinense* from eastern China; CW, *L. chinense* from western China; Ma, Million years ago.

Finally, we applied our framework to the genus *Liriodendron*, which are believed to have undergone significant population reduction throughout their evolutionary history (Chen et al., 2019). Using *K*_s_ distributions, we compared *L. tulipifera* from North America (NA) with *L. chinense* from eastern China (CE) and from western China (CW) (Figure 2A). *Tspecies* inferred the divergence time between NA and CE to be 1.44 Ma with a generation time of 30 years (or 1.21 Ma with a generation time of 20 years), which aligns well with the results obtained from the coalescent-based Bayesian estimation from BPP algorithm (1.38 Ma, Figure 2B; Supplementary Figure S4). Similarly, the estimations for divergence between NA and CW are 1.36 Ma and 1.14 Ma, with a generation time of 30 and 20 years, respectively. In contrast, direct divergence estimates between *L. tulipifera* and *L. chinense* based on mean *K*_s_ values were older, reaching 4.36 Ma (NA vs. CE), tracing back to the warmer Mid-Pliocene period. Additionally, *Tspecies* inferred the ancestral *N*_e_ of *Liriodendron* species to be approximately 5.29 × 10^4^, consistent with the fossil evidence indicating that this genus was once widespread across the Northern Hemisphere (Ian, 2006).

## Discussion

Species divergence has been one of the most central issues in speciation studies. In recent years, with the advancements in genomic evolutionary research, speciation study has been challenged by continuously discovered hybridizations, introgressions, genome duplications, and other complex evolutionary events (Wang and Liu, 2025). An efficient and unbiased solution is hence needed in the inference of species divergence. Our results demonstrate that the variance of *K*_s_ distributions provides a robust signal of ancestral effective population size (*N*_e_), enabling its estimation directly from standard orthologous gene datasets. By leveraging a readily quantifiable property of *K*_s_ distributions, our study offers a scalable framework for integrating coalescent theory into the estimation of species divergence.

Sensitivity analyses confirmed that the *N*_e_–*K*_s_ variance relationship is resilient to variation in sequence length and post-divergence population dynamics. Although accurate substitution rate (*μ*) specification remains necessary, it is always practically required in divergence time estimation, as *μ* is also used as a scaling factor for time calibration. Notably, although *μ* is treated as a categorical variable in our GAM framework, each category corresponds to a narrow interval of *μ* values, and simulations show that variance within categories has only a minor influence on the *N*_e_–*K*_s_ variance relationship (Supplementary Figure S3).

Interestingly, application to *Liriodendron* not only produced divergence estimates consistent with results from coalescent-based Bayesian approaches such as BPP, but also aligned with the climate cooling in the Ice Ages of the early Pleistocene (Lisiecki and Raymo, 2005). Such abrupt climatic shifts are known to drive species range contractions and southward migration, ultimately driving geographic isolation between North American and Asian lineages. These aligned results underscored the population decline from the paleoclimatic and fossil evidence (Ian, 2006).

In addition to estimating species divergence, *K*_s_ distributions from paralogs are extensively used to estimate time for genome duplications (Jiao et al., 2011). Since coalescent theory also applies to the autopolyploids before their diploidization, modeling the relationship between ancestral *N*_e_ and *K*_s_ variance may similarly improve estimates in these contexts. Future work incorporating complex demographic histories, interspecies gene flows, and genome duplication events will further extend the applicability of this model across diverse evolutionary scenarios.

## Data Availability

The datasets presented in this study can be found in online repositories. Tspecies and data used in this manuscript are publicly available under an MIT license at https://github.com/limj0987/Tspecies.git.
