# Supplementary material for: Variance of K s distribution corrects the bias in the divergence caused by the ancestral population size
Source: Front Genet. 2025 Dec 17;16:1725551. doi: 10.3389/fgene.2025.1725551 (PMC12753092; doi:10.3389/fgene.2025.1725551)
Supplement: Supplementary file 1 [file DataSheet1.pdf]

Supplementary materials

Variance of Ks Distribution Corrects the Bias in the Divergence Caused by the Ancestral Population Size

Mi-Jia Li[1,†], Xiao-Xue Li[1,†], Lin-Lin Xu[1,2], Bo-Wen Zhang[1,*]

[1] Ministry of Education Key Laboratory for Biodiversity Science and Ecological Engineering, College of Life Sciences, Beijing Normal University, 100875 Beijing, China.

[2] Department of Microbiome Dynamics, Leibniz Institute for Natural Product Research and Infection Biology (Leibniz-HKI), 07745, Jena, Germany.

[†] These authors contributed equally to this work.

[*] Corresponding information: zhangbw@bnu.edu.cn

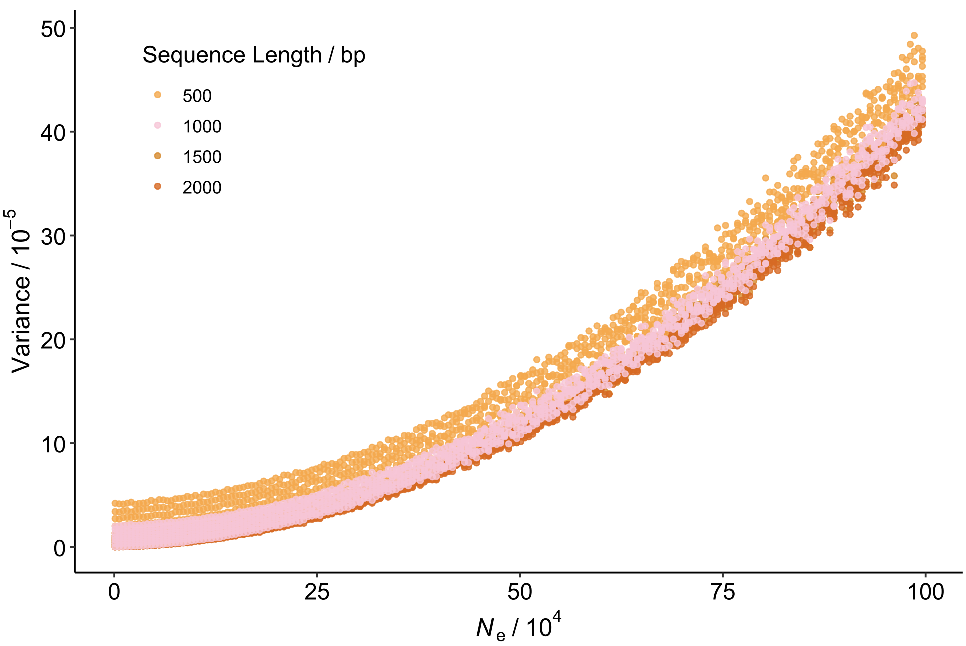

Figure S1. The relationship between N_e and variance of K_s distribution under different sequence length (L). The positive correlation patterns between N_e and K_s variance remained stable across tested L values (500, 1000, 1500, 2000bp). The relative difference suggested potential sequence length effects at shorter L (e.g. $L = 500$bp), while longer lengths ($L \geq 1,000$bp) demonstrated more stable predictions (mean deviation = 12.2%).

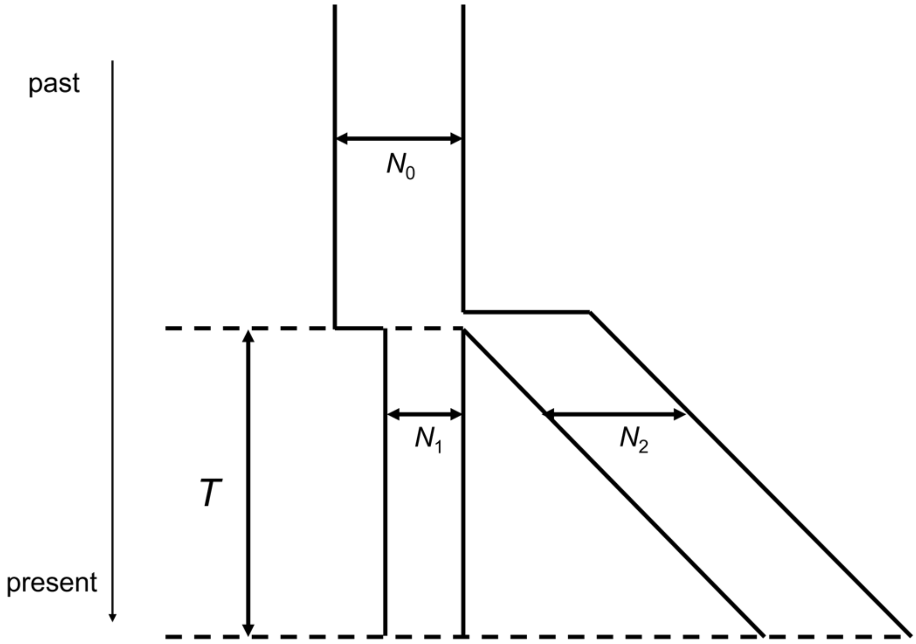

Figure S2. Schematic representation of species divergence scenarios. In simulation, the speciation event transpired T generations ago. Prior to the occurrence of speciation, the effective population size of the ancestor was N_0. Immediately subsequent to the event of speciation, the effective population size of one of the subpopulations underwent a transition to N_1, which corresponded to half, fifth, and tenth of N_0, respectively. The effective population size of another remained constant and equal to that of the ancestor.

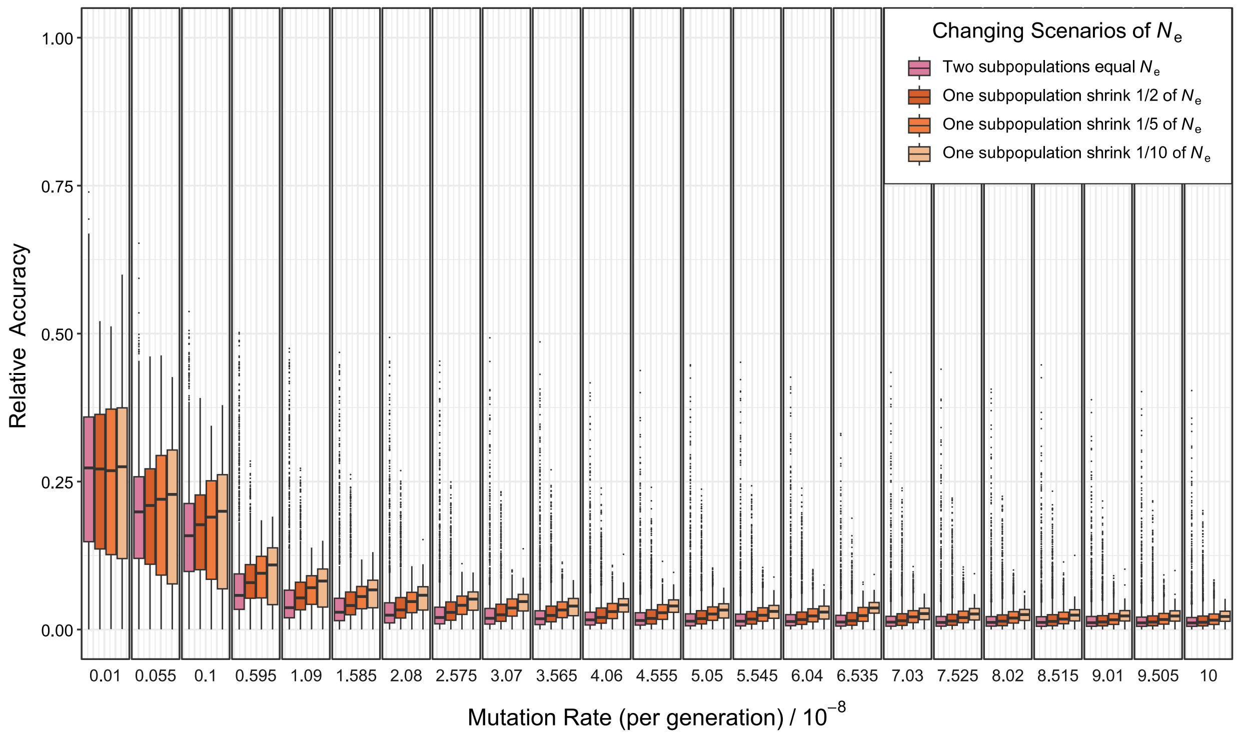

Figure S3. Relative accuracy of species divergence scenarios was calculated using simulated data. Yellow gradients represent scenarios where one subpopulation shrinks to 1/2, 1/5, or 1/10 of the N_e, while the pink bar indicates no change in subpopulation size. We maintained constant subpopulation sizes in this study as the associated impact on accuracy was deemed acceptable (mean relative accuracy = 5.6%), with higher mutation rates (μ) showing improved scenario reconstruction accuracy. The μ gradient was consistent with Fig. 1D, as was the case for the final model (*Tspecies*).

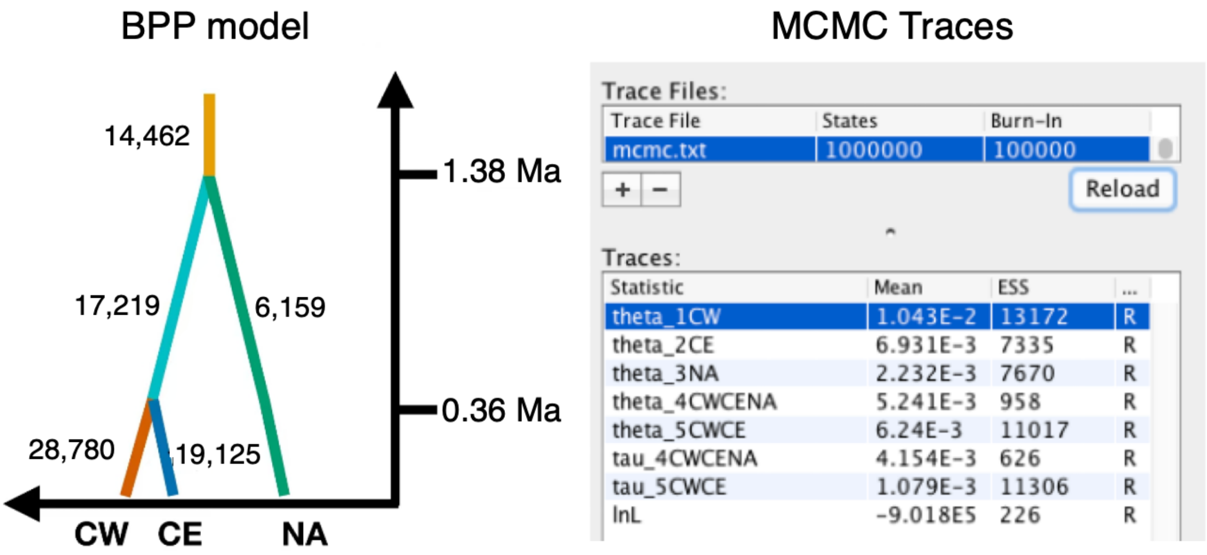

Figure S4. The divergence history of *L. chinense* and *L. tulipifera* estimated by BPP. 1000,000 MCMC runs were performed and the first 100,000 discarded as burn-in. Tau (for divergence time) and Theta (for effective population sizes) parameters were estimated with substitution rate of 3.02×10^{-9} per site per year and generation time of 30 years. The estimates and ESS for each parameter was shown in MCMC traces. NA: *L. tulipifera* from North America; CE: *L. chinense* from eastern China; CW: *L. chinense* from western China.
